# Gellan Gum as a Unique Microbial Polysaccharide: Its Characteristics, Synthesis, and Current Application Trends

**DOI:** 10.3390/gels10030183

**Published:** 2024-03-06

**Authors:** Raghad Abdl Karim Abdl Aali, Shayma Thyab Gddoa Al-Sahlany

**Affiliations:** Department of Food Science, College of Agriculture, University of Basrah, Basrah City 61004, Iraq; pgs.raghad.alhammad@uobasrah.edu.iq

**Keywords:** gellan, *Sphingomonas*, gelrite, hydrogels, application

## Abstract

Gellan gum (GG) is a linear, negatively charged exopolysaccharide that is biodegradable and non-toxic. When metallic ions are present, a hard and transparent gel is produced, which remains stable at a low pH. It exhibits high water solubility, can be easily bio-fabricated, demonstrates excellent film/hydrogel formation, is biodegradable, and shows biocompatibility. These characteristics render GG a suitable option for use in food, biomedical, and cosmetic fields. Thus, this review paper offers a concise summary of microbial polysaccharides. Moreover, an in-depth investigation of trends in different facets of GG, such as biosynthesis, chemical composition, and physical and chemical properties, is emphasized. In addition, this paper highlights the process of extracting and purifying GG. Furthermore, an in-depth discussion of the advantages and disadvantages of GG concerning other polysaccharides is presented. Moreover, the utilization of GG across different industries, such as food, medicine, pharmaceuticals, cosmetics, etc., is thoroughly examined and will greatly benefit individuals involved in this field who are seeking fresh opportunities for innovative projects in the future.

## 1. Introduction

A hydrogel consists of a hydrophilic polymeric network composed of polymers and their solvents. It can absorb significant amounts of solvent and keep it within matrices [1]. Polymers undergo conformational changes in response to variations in solvent conditions like ionic strength and temperature, as described by Tanaka [2]. Hydrogels have extensive applications in the food industry due to their safety features and efficient three-dimensional cross-linked structures, such as fat substitutes, package delivery systems, and edible films [3,4,5]. Carbohydrates and proteins are frequently used as monomers for developing hydrogels. Combining two or more monomers can result in a hydrogel system with enhanced physicochemical properties compared to a single polymer gel [6]. Polysaccharide-based hydrogels have been the focus of significant interest and investigation because of their favorable bioactivity, biocompatibility, and biodegradability. Research has demonstrated that hydrogels composed of polysaccharide-polysaccharide complexes, which exhibit good compatibility, can form a tightly woven interpenetrating network. This can alter the gelation time, improve water retention, and boost the gel’s strength [7]. Enhanced features have been documented for konjac glucomannan-gellan gum, erythritol-curdlan, konjac glucomannan-curdlan, and gellan-xyloglucan complexes [8,9,10,11]. Hence, complexes of polysaccharides could enhance gel properties by leveraging synergistic interactions, rendering them a focal point in the development of gel products.

In recent years, microbial polysaccharides have garnered a great deal of interest due to the amazing functions and vast industrial applications they possess and the fact that they can be produced in a sustainable manner across commercial scales. Methods from the fields of system biology and biochemical engineering may be used to further reduce the costs associated with their manufacture [12]. The ingredients that comprise microbial polysaccharides include both carbohydrate and non-carbohydrate components. They are dependent on the genus of the microorganism that produces the microbial polysaccharide to determine the non-carbohydrate ingredient, which may vary from acetate to pyruvate and even succinate in certain cases [13]. When it comes to microbial polysaccharides, sphingans are distinguished by their tetrasaccharide repeating backbone, which is accompanied by a variety of substitutions on the side chain. Depending on factors such as the linkage pattern, the length of the polysaccharide, the intra-molecular connection, and the polymeric cross-linking with molecules in the surrounding area, the individual sphingan is a rheologically distinct entity [14]. There are considerable differences in the chemistry and functional features of the various sphingans, despite the fact that all of the sphingans are generated by different strains of *Sphingomonas* species [12].

Gellan gum (GG) is an exopolysaccharide secreted outside of the bacterial cell, produced by species from the genus *Sphingomonas*, and it is soluble in water. In 1978, bacteria that produce gellan were found and isolated by the former Kelco division of Merck & Inc. Company, Rahway, NJ, USA. The bacteria were discovered in lily tissue from a natural pond in Pennsylvania. Initially recognized as a substitute gelling agent for agar in solid culture media to support the growth of different microorganisms due to its ability to withstand high temperatures up to 120 °C, it was introduced as a commercial product under the trademark Gelrite GG. It found applications in the pharmaceutical, cosmetic, and food industries [15]. The composition of GG includes three fundamental units: α-L-rhamnose, β-D-glucose, and β-D-glucuronate, in a ratio of 1:2:1. GG possesses transparency, high tensile strength, and a pleasant taste. It exhibits high stability to temperatures and resistance to acidic enzymes. With a molecular weight of up to 500 KDa, it forms a viscous solution when dissolved in aqueous solutions but remains insoluble in ethanol. This compound maintains stability across a broad pH range of 2–10. GG finds extensive applications in the food industry as a thickening agent, binding agent, stabilizer, emulsifier, and gelling agent [16].

GG is derived from various species of *Sphingomonas* bacteria, like *S. pseudosanguinis* and *S. yabuuchiae*, utilizing fat residues like glycerol as a nutritional medium for these bacteria [15]. Huang and colleagues [16] demonstrated the potential of utilizing corn waste to produce GG from *S. paucimobilis* bacteria. The production yield achieved a level of 14 g/L in the production medium. Utilizing molasses and cheese whey as the media, fermentation was carried out to produce GG from *S. azotifigens* bacteria, resulting in a gum with a molecular weight of 890 KDa [17]. GG is utilized in various food industries and is frequently utilized in the production of juices, sweets, beverage powders, jelly, jams, marmalades, vegetable butter (margarine), and yogurt [18,19,20]. In 1990, the gum underwent evaluation by the Scientific Committee for Food of the European Union. It received unconditional approval for use in food due to evidence of cytotoxicity. The gum was assigned the symbol E418 and can be used at concentrations of 0.1–1.0% [21].

After thoroughly examining various aspects in the paragraphs above, the current review paper intends to provide a brief overview of the latest developments in microbial polysaccharides. Moreover, a comprehensive examination of the trends of various facets of GG, such as biosynthesis, chemical composition, and physical and chemical properties, is emphasized. Additionally, the paper focuses on the extraction and purification of GG. Furthermore, a thorough examination of the benefits and drawbacks of GG in relation to other polysaccharides is conducted. Moreover, the utilization of GG in different industries, such as food, medical, pharmaceutical, cosmetics, etc. is critically analyzed. This discussion is expected to greatly influence individuals involved in this field, offering new opportunities for innovative work in the future.

## 2. Microbial Polysaccharides: A Brief Overview

Polysaccharides are commonly located in the vesicles within a cell or are integral components of the cytoplasm. Cell walls play a crucial role in forming the structure, with external polysaccharides aiding in cell adhesion. The structure is a closed capsule that links the cell surface to the body and consists of a sticky substance [22]. These sugars, like xanthan gum and GG, play a crucial role in food production and serve as food additives permitted under US and EU laws and regulations. Many microorganisms naturally produce these sugars and release them outside of the cells. Some of these microorganisms can produce significant amounts exceeding 40 g/L, suitable for applications in the food and pharmaceutical industries [23]. Polysaccharides derived from microorganisms possess unique characteristics that render them more useful than plant-based polysaccharides. They can be produced quickly, typically within a few days, as opposed to plant-derived polysaccharides, which may take months or even years to produce. Additionally, microorganism-derived polysaccharides are available year-round, unlike plant sugars, which are only available during certain seasons. Industrial byproducts like hydrocarbon residues and glycerol can serve as carbon sources for producing these sugars from microorganisms. Despite the numerous advantages of these polysaccharides, the production cost remains a significant limitation, which hinders their widespread use. Large fermenters and nitrogen and carbon sources are required for their production [22]. Microorganisms produce polysaccharides with high molecular weights. These polysaccharides are produced by various microorganisms like bacteria, fungi, and algae. They serve as a source of energy, carbon storage, and building materials for cell walls. Some polysaccharides, like glycogen and chitin, are found inside cells, which help in cell wall formation and protection. Others are secreted outside of cells to adhere to surfaces, while some form capsules around cells for protection and adhesion [24]. The polysaccharides found in capsules are typically produced by pathogenic bacteria. This aids in binding bacteria to infected cells. Exopolysaccharides, on the other hand, are resistant to environmental conditions, assist in adhering to external surfaces, and serve as water and carbon storage for the producing cells [25]. Moreover, microbial polysaccharides consist of monosaccharide units and may also include non-carbohydrate components like phosphate, pyruvate, succinate, and acetic acid. They are categorized into homopolysaccharides, composed of a single basic unit like glucose in cellulose, and heteropolysaccharides, which have various sugar units and non-carbohydrate components like xanthan gum, hyaluronic acid, and GG (Table 1).

Polysaccharides that are derived from microorganisms (microbial polysaccharides) and those sourced from plants display various distinctions, such as their structures, origins, roles, and practical uses. A comparison of microbial polysaccharides and plant polysaccharides is presented in Table 2, highlighting their key differences. Moreover, microbial and plant polysaccharides have similar uses, but their distinct structures and origins lead to differences in their characteristics and functions. Choosing between microbial and plant polysaccharides relies on the specific application needs and desired traits in the final product.

## 3. Gellan Gum: An Overview of the Trends

In 1978, Merck in the US identified polysaccharides generated extracellularly by *Pseudomonas elodea*; these compounds were codenamed S60 or PS60 [12]. The general name for these substances is GG. Isolated from the Elodea plant, the bacterium responsible for producing this gum is known as *P. elodea*. The strain was identified as *S. paucimobilis*, a proteobacteria, in 1994 as a result of more extensive genetic and biochemical research [43]. In 1988, GG was found to be safe to use as a food additive in Japan after a battery of tests that established its non-toxicity. The United States Food and Drug Administration gave the green light to GG in 1992 so that it may be used in food. The European Union classifies GG as an E418 food additive [12].

### 3.1. Chemical Composition of Gellan Gum

**Figure 1:**
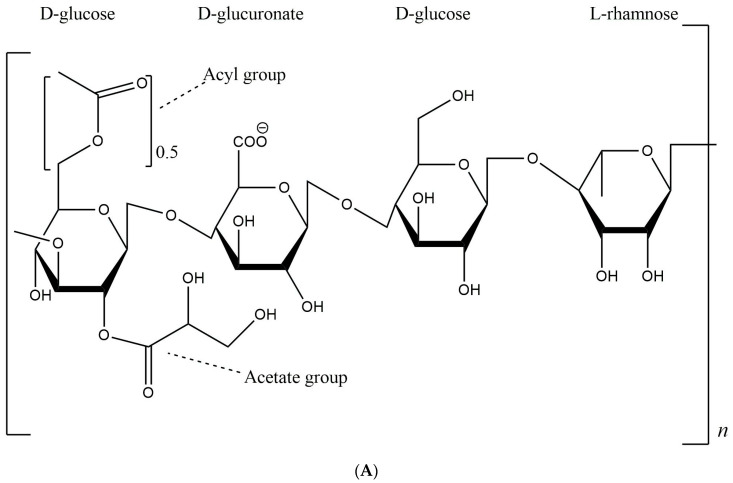

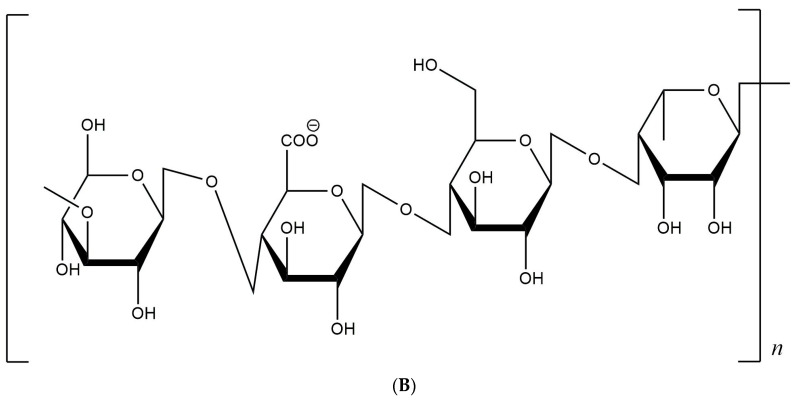
Chemical structures of gellan gum: (**A**) natural high-acyl gellan and (**B**) low-acyl gellan.

GG is a linear polysaccharide that contains negative charges, known as polyanions. It is composed of four basic units: two β-D-glucose molecules, one β-D-glucuronate molecule, and one α-L-rhamnose molecule. Glucose accounts for 60% of the polymer composition of GG, with rhamnor sugar and glucoronate each comprising 20% [43]. The GG that is produced comes in two varieties: the natural type, recognized for its high acyl content and referred to as high acyl GG. There is a second type known as low acyl, where the acyl group is removed, specifically, low acyl GG (Figure 1). One key distinction between these two varieties lies in the existence of two acyl groups, glycerides and acetates. During the purification process of natural GG, the acyl group can be eliminated using a hot base solution, which then impacts the rheological properties [44]. There are two variations of GG utilized in food and pharmaceutical applications, chosen based on the product type and desired hardness.

### 3.2. Physical and Chemical Properties of Gellan Gum

GG is a complex exopolysaccharide with a molecular weight of approximately 500 KDa. It exhibits good solubility in water at temperatures exceeding 40 °C (Table 3). Research has indicated that by utilizing X-ray diffraction, GG possesses a three-sided parallel double helix structure [45]. The hydrogen bonds create the double helix structure by linking the hydromethyl groups in the glucosyl units with the carboxylate groups along the chain to form the polymer. The composition is influenced by the concentration of the polymer added to the aqueous solutions, temperature, and the existence of positively charged compounds [46].

Miyoshi and Nishinari [47] demonstrated that GG can create stable and transparent gels at pH levels between 3 and 5. The formation of crystals is influenced by the existence of positive charges within the solution. By raising the temperatures and disrupting the crystalline structure, the compound can revert to its initial state. These are the key rheological characteristics of GG. Key rheological characteristics include yield stress and significant shear thinning behavior, as highlighted in studies by Sworn et al. [48], Farrés and Norton [49], and Ghebremedhin et al. [50]. Furthermore, exploring the relationship between yield stress and the initiation of non-linear time-dependent rheological properties in GG fluid gels containing 0.2 wt% GG and 0.2 wt% Ca^+2^ as gel formation promoters. This involves conducting creep-recovery-creep tests for shear stresses ranging from 2 to 6 Pa while ensuring the elimination of shear history effects [51]. The presence of these properties may be tuned via the use of processing and formulation parameters, which offers a great deal of flexibility for the purpose of tailoring the material to specific industrial applications [48,49,50]. Furthermore, Giavasis and colleagues [52] discovered that GG remains stable even at high temperatures. At 90 °C, it creates a robust gel in contrast to xanthan gum, which experiences a 74% weight loss under similar conditions. GG has a melting point of 100 °C or higher, which varies based on the presence of positively charged elements. This enhances bonding between the gel units, increasing its temperature resistance. As a result, GG can serve as a substitute for thickeners and stabilizers in food applications. Zia et al. [53] highlighted the key properties of GG, emphasizing its crystallization capability, resistance to shear force, flexibility, biodegradability, and thermal and acid stability.

### 3.3. Biosynthesis of Gellan Gum

**Figure 2:**
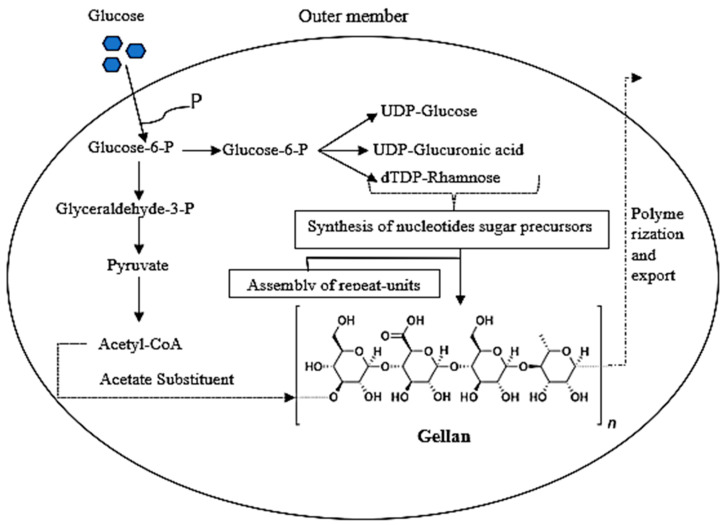
The metabolic pathway of *Sphingomonas* bacteria to produce gellan gum.

The production of GG relies on three fundamental and consecutive steps, which entail activating the sugar units that form the adhesive within the cells. In the next step, the basic units are put together into quadruplex repeats and connected to the inner cell membrane. This is followed by the final step of moving this repeat outside the cell once the polymerization process is complete, with the transfer happening across the outer membrane [14]. The bacteria that produce GG consume glucose through either the Embden-Meyerhof metabolic pathway or the pentose-phosphate pathway in the absence of the phosphofructokinase enzyme, which is the initial step in the glucose catabolism process in this pathway (Figure 2). In a study by Giavasis et al. [52], it was demonstrated that the lack of the enzyme glucose-6-phosphate dehydrogenase in the cell does not impact glucose consumption or GG production, as this enzyme is not part of the glucose metabolism process. The primary enzyme involved in glucose breakdown during the production of GG is either free glucose dehydrogenase or the glucokinase enzyme. Manufacturing GG starts with converting glucose into glucose-6-phosphate, which then leads to the formation of the uridine diphosphate and thymine diphosphate units along with the phosphorylated uridine diphosphate base. It plays a crucial role in the creation of glucose and glucuronic acid units, while thymine diphosphate is in charge of producing rhamnose sugar. These three sugars are connected to form quaternary units, which are then polymerized to create GG [43].

### 3.4. Extraction and Purification of Gellan Gum

Organic solvents, such as ethanol and IPA, are commonly used in the industry for precipitating GG from broth. Nevertheless, it does not ensure the necessary purity and, therefore, needs additional purification. When GG is anionic, it can be precipitated by protonating it with the addition of acids. The protonated form of GG has a low affinity for the solvent, causing it to precipitate out easily with a lower solvent volume [54,55]. Using organic solvents for precipitation to eliminate impurities is a common practice in the commercial production of GG. Nevertheless, the increased need for solvent volume renders the process costly and ecologically detrimental. By using electrolytes like CaCl_2_, NaCl, and KCl, one can decrease the amount of solvent needed by 2.5–3.0 times, resulting in cost savings [56]. The strength of IPA or ethanol used is a crucial parameter affecting the precipitation yield and quality of GG. It is recommended to use a strength of at least 90% to obtain fibrous, off-white gellan that is free of impurities.

Every polysaccharide requires specific growth conditions and environmental factors that can be adjusted to enhance the yield of production. When producing gellan, a basic media with a carbon source, nitrogen source, and inorganic salts can be used. Alternatively, a more complex media with added vitamins can boost cell growth [57]. When compared to other hydrocolloids, GG production is more costly. Therefore, there are numerous studies in the literature suggesting the use of industry byproducts to lower biosynthesis costs [16,58,59]. For instance, Banik and colleagues utilized the Plackett-Burman design to investigate the impact of different nutrient supplements on gellan production. They opted for molasses, a sugar industry byproduct, as the carbon source instead of glucose, sucrose, lactose, or starch [59]. Raghunandan and colleagues utilized biodiesel-derived waste glycerol as the primary carbon source for gellan production, resulting in high yields for both bacterial strains (*S. pseudosanguinis* and *S. yabuuchiae*) of 51.6 and 52.6 g/L [15]. In this study, gellan was produced at a reduced cost while successfully conducting the bioremediation of waste glycerol. In a study, Fialho and colleagues examined the impact of glucose, lactose, and cheese whey on the production of gellan, a biopolymer [60]. They discovered that using various carbon sources resulted in gellan with distinct characteristics, such as differences in the acyl substitution level, polymer rheological properties, and susceptibility to degradation. However, the yield achieved for diluted cheese whey was 7 g/L, comparable to the yield for lactose, which was 9 g/L.

According to Fialho et al. [14] and Huang et al. [16], yeast extract, peptone, or ammonium nitrate are the most common complex nitrogen sources, whereas sodium nitrate and yeast extract are inorganic sources. Researchers have explored byproducts, such as corn steep liquor and soybean pomace, as a cost-effective nitrogen source for gellan production [16,61]. According to Huang et al. [16], corn-steep liquor showed promise as an industrial tool after replacing peptone, yeast extract, and soybean ingredients in gellan synthesis. In addition, the GG synthesis by *S. paucimobilis* NK2000 was examined with soybean pomace instead of bacto-peptone. The researchers discovered that using soybean pomace as a nitrogen source resulted in more gellan production compared to bacto-peptone [61]. This suggests that soybeans can be utilized to create GG, which can help to reduce environmental contamination from agro-industrial byproduct disposal.

An additional crucial factor in gellan synthesis is temperature, which is typically maintained at a temperature of 30 °C during the majority of fermentation processes [43,58,61]. The highest yield is produced at temperatures between 20 and 25 °C, and beyond 30 °C, the yield drops dramatically [62]. When it comes to cell proliferation and gellan synthesis, pH is a key factor. Researchers have investigated the impact within a range of 4 to 10, finding that the highest yield of gellan and biomass was attained at a pH of 7.0 [43,58]. Researchers have suggested that gellan production should be conducted within a pH range of 6.5 to 7.0 [52]. When examining the agitation rate, it was noted that insufficient agitation failed to homogenize the broth, while high stirring rates (600–800 rpm) resulted in the development of a stagnant layer and inconsistent broth, impacting the heat and mass transfer. According to Giavasis et al. [52], the gellan broth should be mixed at a speed of 250 rpm.

The recovery of GG following successful fermentation is an important phase for which two options are available. Kang and co-workers [63] developed the first approach that produces GG with divalent cations, whereas Manna and colleagues [64] described the second method that produces GG with monovalent cations. The primary distinction between the two approaches is that the second one uses centrifugation instead of filtering. In Kang et al. [63], two volumes of 99% isopropanol were used to precipitate the filtrate to obtain a deacetylated and clarified gellan. A cleared and deacetylated gellan was obtained by precipitating it with four volumes of propanol [64]. In Giavasis et al. [52], the gellan that was recovered using both procedures was then dried at 55 °C for 1 h. According to Jin and colleagues [61], GG can be refined by first dissolving it in deionized water and then washing it with acetone and ether to remove any isopropanol precipitate. The next step is to dialyze it against the deionized water for two or three days. To re-obtain GG, the mixture is lyophilized [61]. Additionally, a study was conducted to assess various methods for recovering and purifying GG from the fermentation broth [65]. The gellan obtained from the various procedures was examined using FTIR and NMR. The most optimal outcomes in terms of purity were obtained through the following steps: filtration, rinsing with acetone and ether, dissolving the permeate with distilled water, and precipitating with acetonitrile [65].

## 4. Advantages and Disadvantages of Gellan Gum Compared to Other Polysaccharides

GG is a versatile hydrocolloid with applications that can be compared to other polysaccharides like agar, carrageenan, and xanthan gum. Generally speaking, GG offers the benefit of forming gels at low concentrations and being thermoreversible, which enables the development of distinctive textures. It exhibits resistance to syneresis, ensuring the stability of gels over an extended period. GG exhibits excellent clarity and film-forming characteristics. When it comes to contracts, one drawback of GG is that it might need higher concentrations for specific uses in comparison to other gelling agents. The presence of specific ions can impact the texture of GG gels. GG is a less recognized option and might come with a higher price tag compared to other hydrocolloids. A comparison of the benefits and drawbacks of using GG versus other polysaccharides is presented in Table 4. When choosing GG over other polysaccharides, various factors come into play, such as the intended texture, stability, processing conditions, and cost. Every hydrocolloid has its own advantages and disadvantages, so the decision should be selected based on the desired functionality and sensory characteristics of the end product. Simply put, the selection of GG, agar, carrageenan, or other polysaccharides is based on the particular needs of the application. GG is well-suited for situations requiring a thermoreversible gel with strong stability, commonly found in various food and pharmaceutical products. Every polysaccharide comes with its own set of advantages and disadvantages, so choosing the right one depends on factors like the desired texture, stability, processing conditions, and cost.

## 5. Applications of Gellan Gum

### 5.1. Food Applications

GG is a food additive that has been utilized for an extended period because of its functional properties. Currently, there is a growing interest in this field because of the advancements in new materials and the enhancement of food systems to improve their rheological properties [73]. GG was utilized in colloidal aqueous solutions due to its capacity to produce gelatinous substances at low concentrations and establish a network in contrast to other polycarbonates employed in foods (Table 5). Adjusting the gum, like eliminating the acetyl group, led to the production of gelatinous materials with unique physical characteristics, enabling its utilization in various food product models [74].

In various industries, polygons found in foods are combined with other elements to eliminate certain undesirable characteristics. For instance, GG has low mechanical strength, so when combined with pullulan, it resulted in a product with favorable sensory properties and distinctive rheological characteristics [76]. In a study by Kanyuck et al. [77], it was demonstrated that combining GG with maltodextrin resulted in the formation of a robust, intertwined network. This blend was utilized in low-fat items as a substitute for fat while also providing a sensation of satiety. In a study by Sapper et al. [78], it was found that incorporating thyme oil with GG in creating a film for fruit preservation resulted in the development of an edible composite film. This film extended the preservation period by reducing evaporation and inhibiting the growth of microorganisms.

Recently, it was discovered that GG can act as a stabilizing agent and also preserve volatile oils in coated fruits. It can improve the percentage of ascorbic acid and antioxidant activity, enhance preservation processes, and extend the storage life of highly perishable fruits, ultimately prolonging their stay in the market [79]. GG is a cost-effective multivitamin that is ideal for use in the production of sweets and jams due to its high crystallinity and its capacity to regulate the product’s moisture. Not only is the gel colorless and has a high melting point but sweets can also be stored at room temperature. Research has shown that the optimal concentration for sweets is 0.3%. Foods with GG can retain their shape and appearance for an extended period due to its water-absorbing properties [80]. In a study, Kong et al. [81] discovered that incorporating 0.1% of low-acyl GG into soybean milk yogurt resulted in the development of a stable gel network with increased viscosity, enhanced water retention capacity, and a smoother texture in comparison to the control group. Thus, GG is regarded as a stabilizer. It is appropriate for this product while also enhancing its sensory characteristics. In a study by Mongkontanawat and Khunphutthiraphi [82], it was found that incorporating 10% GG into sprouted black rice for vegetable yogurt resulted in a soft texture and good sensory appeal. This addition did not impact the moisture, protein, or fat content but also increased the levels of antioxidants and lactic acid bacteria. The study demonstrated significant toxicity toward breast cancer cell lines when compared to yogurt produced from sprouted rice without added GG [82]. This offers the opportunity to create nourishing and wholesome meals for adults and elderly people.

#### Production and Utilization of Edible Films

Edible films developed from GG have become increasingly popular in food-related uses because of their versatility and advantageous characteristics [12,52]. These films are thin layers of material that can be ingested with food, offering different functionalities. An overview of the production and applications of edible films using GG in the food industry is provided in Table 6 and Table 7. Utilizing GG in edible films presents a sustainable and functional method for food-related uses, delivering advantages like decreased waste, enhanced preservation, and improved sensory experiences for consumers [12,15].

### 5.2. Medical and Pharmaceutical Applications

The plant’s characteristics, its resistance to temperature, and its capacity to expand and be trimmed all play a role in the various functions of GG. Consequently, it has been incorporated into a diverse range of medicinal formulations (Table 8). Aside from being utilized in gene transfers for certain health purposes, it has also been employed to develop ointments to treat dental and ocular conditions [97]. To explore the gelatinous properties, Bajaj et al. [43] mixed GG with the antibiotic ciprofloxacin. Ciprofloxacin is an antibiotic that effectively targets both gram-negative and gram-positive aerobic bacteria. According to these results, the mixing process led to a longer duration of medication release in the body, enhancing the medicine’s effectiveness against specific bacteria. Dev et al. [12] showed that a capsule produced with GG consisted of an ionic solution in the presence of water containing calcium ions. This solution formed a three-dimensional network of GG gel by creating intersections in the polymer chain caused by the presence of these ions. This led to the creation of a capsule that could be used to deliver the drug to humans.

The fact that GG exhibits poor mechanical strength, brittleness, and a low degree of crystallinity when it is combined with pharmaceutical and medicinal ingredients is one of the challenges that is linked with the development of medications utilizing GG. By dividing the GG chains into smaller parts with the help of an oxidizing agent called sodium periodate, a study was carried out with the purpose of finding a solution to this issue. This led to the appearance of hardness in the gel, which in turn led to an increase in the amount of time that the drug was available within the body. Additionally, the use of GG as a binder resulted in an increase in the number of viable bacteria cells [104]. Silva et al. [105] observed that GG is an effective carrier biomaterial that also improves cell adhesion. Furthermore, they stated that the polymer was modified by including a peptide that was obtained from fibronectin, which is a glycoprotein that is formed outside of the cell cells. Additionally, in comparison to unmixed GG, this combination resulted in the multiplication of neural stem cells, and the results of this research demonstrated that it was successful in treating damage connected to the spinal cord. GG has been applied in the field of tissue engineering as well. As per research conducted by Vasile et al. [106], GG hydrogels were utilized as scaffolds for the regeneration of different tissues like bone and cartilage. The hydrogel’s capacity to replicate the extracellular matrix and offer mechanical support to the cells positions it as a prime choice for tissue engineering purposes. Aside from its function in drug delivery and tissue engineering, GG has also been utilized in wound healing. Alharbi et al. [107] explored the application of GG-based hydrogels in treating chronic wounds. The researchers discovered that the hydrogel supported wound healing by creating a moist environment that helped cells migrate and boosted angiogenesis. Moreover, GG has been investigated for its promise in the realm of regenerative medicine. An investigation conducted by Ghandforoushan et al. [108] showcased the application of GG hydrogels in transporting stem cells. The hydrogel created an optimal microenvironment for the stem cells, improving their ability to survive and differentiate, ultimately boosting their therapeutic capabilities. Furthermore, GG has been used in creating controlled-release dosage forms. A study conducted by Carrêlo et al. [109] reported the development of GG-based microparticles for the sustained release of drugs. The microparticles demonstrated a controlled release profile that enabled extended drug release and enhanced therapeutic effectiveness.

#### Responsive Systems for Biomedical Applications

GG, a highly versatile hydrocolloid, has been receiving increased interest in the biomedical sector because of its exceptional properties and potential uses in developing responsive systems [110,111]. There have been several cutting-edge advancements in the use of GG to develop responsive systems in the field of biomedical applications (Table 9). The latest developments in the use of GG in various biomedical fields demonstrate its ability to advance drug delivery [107,110,111,112], tissue engineering [113,114], wound healing [115,116], and diagnostics [111,117]. The responsive nature of GG enables the development of specific systems that react to particular physiological signals, enhancing their efficiency and flexibility in different biomedical scenarios.

### 5.3. Applications in the Cosmetics Industry

Microbial polysaccharides are utilized in cosmetics for their unique physical and chemical properties as well as their biological activities. GG can be utilized in cosmetics due to its ability to enhance viscosity at low concentrations. Polysaccharides like hyaluronic acid, bacterial cellulose, levan, and GG play a crucial role as active ingredients. GG aims to enhance water retention and alter rheological characteristics [129]. GG is a key component in numerous cosmetics, serving to enhance viscosity and act as a stabilizer for certain emulsions applied to and left on the skin. It has been utilized in concentrations ranging from 0.3% to 0.5%. It can also be utilized in the production of toothpaste at concentrations ranging from 0.025% to 0.25%. These minimal amounts provide durability to the product even at temperatures as high as 60 °C, along with its thin texture and uniformity [57]. In a study by Iurciuc et al. [130], it was discovered that GG has positive effects on cosmetics, particularly in hair moisturizing and sun protection products. When GG is applied to the skin, it provides a pleasant sensation to the user. It was combined with xanthan gum to enhance the effectiveness of hair care products, being utilized at a concentration of 0.2%.

### 5.4. Biological Applications

GG, derived from bacteria, has been utilized as a substitute for agar in fields for cultivating various microorganisms. It has been introduced to the market as Gelrite because certain microorganisms have specific enzymes that can break down agarose into the compound 3,6-anhydro. L-galactose and D-galactose are indicated in reference [131]. GG was developed under a different brand name, phyta gel, and is commonly utilized as a replacement for agar in agricultural media. It also helps in solidifying the agricultural media for growing tissue plants [132]. GG was utilized for the separation of DNA components post-extraction from various sources through electrophoresis. It acts as a gel with pores, enabling the passage of these components [133].

## 6. Potential Future and Research Possibilities

Exploring research possibilities in microbial polysaccharides covers a wide range of fields, such as microbiology, biotechnology, food science, pharmaceuticals, and materials science. Based on current findings from the literature review, GG is a microbial polysaccharide produced by the bacterium *Sphingomonas elodea*. It has found extensive applications in the food industry for its gelling, stabilizing, and thickening properties. After conducting a literature review, we propose potential future research opportunities and developments (Table 10) related to GG based on its properties and potential applications. These research opportunities showcase the wide array of possibilities in the field of GG microbial polysaccharides, with potential implications for industry, health, and the environment. Researchers and scientists have the freedom to select subjects that align with their passions, knowledge, and desired societal contributions. Furthermore, researchers and scientists in these fields could offer valuable insights into broadening the uses of GG and enhancing its manufacturing methods. As research on GG microbial polysaccharides progresses, the possibilities ahead will offer extensive potential for imagination and influence in various industries. Therefore, researchers and scientists are encouraged to delve into these areas according to their interests, expertise, and the changing needs of society.

## 7. Concluding Remarks

With its distinctive natural properties, GG is extensively utilized in a range of industries, including food, cosmetics, and pharmaceuticals. Studies indicate that microbial production methods may offer a more economical alternative to chemical synthesis. It is crucial as a food hydrocolloid and demonstrates promise as a prebiotic for developing cutting-edge functional food components. Nevertheless, further investigation is required to demonstrate its efficacy as a food supplement. It is essential to establish international standards for prebiotics and supplements such as GG and conduct additional research to investigate their potential therapeutic applications. The distinctive natural characteristics of GG have proven its worth in a range of industries, such as dairy, food, cosmetics, and pharmaceuticals. Moreover, investigating the advantageous production of GG from more affordable sources through microbial cells could potentially better meet industrial needs compared to chemical synthesis. Further investigation is required to demonstrate GG’s efficacy as a food additive, whether as a stabilizer, viscosity enhancer, or texture enhancer. Legislation and regulatory rules for prebiotics and innovative dietary supplements like GG vary significantly from one country to another, requiring the need to address this diversity and establish consistent international standards. Furthermore, the intriguing prebiotic and immunostimulatory characteristics necessitate further in vitro and in vivo investigations to determine the potential of this molecule in treating severe diseases.

## Figures and Tables

**Table 1:** Polysaccharides produced by microorganisms.

Producing Microorganisms	Type of Sugar Produced	Reference
*Agrobacterium tumefaciens*	Succinoglycan	[26]
*Gluconacetobacter*, *Sarcina Agrobacterium*, *Rhizobium*	Cellulose	[27]
*Agrobacterium leguminum*	Curdlan	[28]
*Leuconostoc dextranicum*	Glucan	[29]
*Leuconostoc*, *Streptococcus*, *Weissella*, *Pediococcus Lactobacillus*	Dextran	[30]
*Pseudomonas aeruginosa*	Algins	[31]
*Acinetobacter* sp.	Emulsan	[32]
*Sphingomonas paucimobilis*	Gellan	[33]
*Streptococcus equisimilis*, *S. Pyogenes*, *S. thermophilus*, *S. equi*	Hyaluronic acid	[34]
*Acetobacter*, *Bacillus*, *Brenneria*, *Geobacillus*, *Halomonas*, *Lactobacillus*, *Zymomonas*, *Saccharomyces*	Levan	[35]
*Aureobasidium pullulans*, *Cytaria* spp., *Teloschistes flavicans*, *Rhodototula bacarum*, *Cryphonectria parasitica*	Pullulan	[36]
*Streptococcus mutans*	Mutan	[37]
*Xanthomonas* spp.	Xanthan gum	[38]

**Table 2:** Some of the most important differences between plant polysaccharides and those produced by microorganisms [39,40,41,42].

Features of Polysaccharides	Microorganism Polysaccharides	Plant Polysaccharides
Source	Produced by microorganisms like bacteria, yeast, and fungi. Some examples are xanthan gum (bacterial), dextran (bacterial), and pullulan (fungal).	Derived from botanical sources. Some examples are cellulose, hemicellulose, pectin, and starch.
Structure	They frequently exhibit a more intricate and varied composition. Illustrations feature branched structures in xanthan gum and linear structures in dextran.	Usually exhibit well-defined and consistent structures. Illustrations consist of the straight arrangement of cellulose and the intricate arrangement of pectin.
Function	Frequently produced by microorganisms for functions like protection, attachment, or energy retention. Applications in industries involve serving as thickeners, stabilizers, and gelling agents in the food and pharmaceutical sectors.	Plants perform a range of functions, including providing structural support (cellulose), storing energy (starch), and maintaining cell wall integrity (hemicellulose and pectin). They are applied in a variety of industrial settings, such as food, pharmaceuticals, and paper production.
Production Method	Produced via fermentation techniques with microorganisms in controlled environments.	Derived from plant tissues using a combination of physical and chemical methods, which may include breaking down cell walls to release polysaccharides.
Solubility	Certain microbial polysaccharides, such as xanthan gum, exhibit water solubility and produce viscous solutions.	The solubility of plant polysaccharides differs from one another. As an illustration, starch does not dissolve in cold water, whereas pectin does.
Gelling Properties	Certain microbial polysaccharides like GG demonstrate gelling properties and can produce firm, flexible gels.	Agar and pectin, plant-derived polysaccharides, are recognized for their gel-forming properties and are widely utilized as gelling agents in the food and pharmaceutical industries.
Applications	Commonly utilized in the food, pharmaceutical, and cosmetic sectors for their thickening, stabilizing, and gelling properties.	Applied in a wide range of industries, such as food (for thickening and gelling), pharmaceuticals, textiles, and paper production.

**Table 3:** Physical, chemical, and microbial properties of the resulting gellan gum.

Tests	Property	Value
Physicochemical	Molecular weight	500 KDa
	Appearance	Yellowish white powder
	Functional use	Thickener and stabilizer
	Solubility	Dissolved in water, forming a viscous solution, insoluble in ethanol and other organic solvents
	Weight loss	No more than 15% when using a temperature of 105 °C for 2.5 h
	Lead	No more than 2 mg/kg
	Nitrogen	No more than 3%
Microbial	Total bacterial count	No more than 1 × 10^4^ CFU/g
	*E. coli*	There was no growth
	*Salmonella*	There was no growth
	Yeasts and molds	No more than 5 × 10^2^ CFU/g

**Table 4:** A comprehensive evaluation of the benefits and drawbacks associated with the use of gellan gum in comparison to other polysaccharides [53,66,67,68,69,70,71,72].

Features	Critical Remarks
Benefits	
Thermoreversibility	GG develops thermoreversible gels, which can solidify when cooled and return to a liquid form when heated. This feature is advantageous in scenarios where precise temperature management is crucial, enabling the creation of distinct textures in food and pharmaceuticals.
Low concentration requirement	GG can produce gels at lower concentrations in comparison to certain other polysaccharides, such as agar. This can offer benefits in terms of cost-effectiveness and sensory impact.
Resistance to syneresis	GG gels typically exhibit resistance to syneresis, the phenomenon where a liquid is released from a gel. This characteristic plays a role in maintaining the stability and visual appeal of products that contain GG as time passes.
Clarity	GG gels are known for their excellent clarity, rendering them ideal for uses in applications that require a transparent or semi-transparent look, like specific desserts or beverages.
Film-forming ability	GG possesses film-forming properties, which render it ideal for use in scenarios where thin, pliable films are needed, like in edible films for food encapsulation.
Drawbacks	
Texture sensitivity to ions	The texture of GG gels can be affected by the specific ions and their concentrations in the formulation. Its sensitivity could restrict its use in specific circumstances.
Limited familiarity	When compared to other polysaccharides, such as agar or xanthan gum, GG might not be as familiar or commonly utilized in specific industries or sectors, potentially affecting its adoption and accessibility.
Cost	GG might have a higher price compared to certain other hydrocolloids, which could be an important factor to keep in mind when cost is a crucial consideration in formulations.

**Table 5:** Typical food applications of gellan gum.

Main Food Field	Representative Food Product	References
Beverages	Beverages with jelly and fruit	[74]
Sugar	Starch, jelly, stuffing, and candy floss	[61]
Jam	Low-heat jam, synthetic jam, and bread stuffing	[62]
Synthetic food	Synthetic fruits, synthetic vegetables, and synthetic meat	[18,19]
Water-based gel	Dessert gel and decorative jelly	[47]
Pie stuffing and pudding	Fast-food dessert, tinned pudding, pre-cooked pudding, and pie stuffing	[75]
Pet food	Tinned meat segment and gel pet food	[71]
Sugar coating and sugar frost	Sugar coatings for cakes and tinned sugar frost	[75]
Milk products	Ice cream, jelly milk, yogurt, and frozen milk	[60]

**Table 6:** Production of edible films using gellan gum [83,84,85].

Aspects of Edible Film Production	Remarks
Ingredient selection	GG has been chosen as the primary hydrocolloid for film formation. Additional components can be incorporated to improve the characteristics of the film, including plasticizers like glycerol and sorbitol, antimicrobial agents, antioxidants, or flavorings.
Solution preparation	GG is commonly dispersed in water or a water-based solution. Next, the solution is heated to fully hydrate and dissolve the GG.
Film formation	Once the solution is ready, it is poured or cast into a mold or onto a flat surface to create a thin layer. The film-forming solution can be dried using techniques such as air drying, hot air drying, or freeze-drying based on the desired properties of the film.
Control of film properties	By adjusting the concentration of GG and other additives along with the drying conditions, it is possible to control the properties of the edible film, including the thickness, transparency, and mechanical strength.

**Table 7:** Versatile applications of gellan gum-based edible films across various fields of food.

Multiple Applications within the Food Sector	Remarks	References
Food packaging	Edible films composed of GG are used as packaging for a variety of food items. These films serve as protective shields that block out moisture, oxygen, and other environmental elements, ultimately prolonging the shelf life of perishable items.	[86]
Coatings for fresh produce	Edible films developed from GG are suitable for coating fresh fruits and vegetables. The films aid in decreasing water loss and preserving freshness and can also be used to transport additional nutrients or preservatives.	[87,88,89]
Encapsulation of bioactive compounds	GG films are ideal for encapsulating and protecting bioactive compounds like antioxidants, vitamins, or antimicrobial agents. This enables a regulated release of these substances within the food matrix.	[75,90,91]
Flavor films	GG-based films have the capability to incorporate flavors or aromas, offering a distinctive and personalized sensory encounter when utilized as coverings for candies, confections, or other flavored food items.	[92,93]
Edible strips and wrappers	GG films can be shaped into strips or wrappers that are convenient to use and eat. This is especially beneficial for products that require a thin, dissolvable layer, like single-serving condiment packets.	[94]
Improvement of texture	Edible films composed of GG have the potential to enhance the texture of specific food products, resulting in a more pleasing mouthfeel and improved crispiness.	[95,96]
Other innovative uses	Exploring GG-based edible films in different innovative applications such as edible food labels, decorations, and interactive food experiences.	[41]

**Table 8:** Examples of gellan gum-based micro and nanoparticulate systems.

Drug/Application Formulation	Fabrication Procedure	Most Important Type	Results	Reference
Model microgels	Ionotropic gelation with CaCl_2_ or KCl, coating with chitosan	Microgels	Good stability in aqueous media except for KCl-crosslinked microgels; the particles were stable in gastric conditions; chitosan-coated microgels were less susceptible to degradation in intestinal fluid	[98]
Prednisolone, paclitaxel/cancer	Self-assembly	Nanogels	Prednisolone acted as a hydrophobic moiety in nanogel self-assembly; increased cytotoxic efficacy towards different cancer cell lines	[99]
Curcumin/cancer	Polyelectrolyte complexation	Nanogels	Prolonged curcumin release; good hemocompatibility and non-toxicity	[100]
Piroxicam/non-melanoma skin cancers	Self-assembly	Nanogels	Nanogels enhanced drug retention in the epidermis; nanogels permeated across the stratum corneum and released the drug in the viable epidermis	[101]
Probiotic bacteria/gut microbiota dysbiosis	Ionic crosslinking with CaCl_2_, freeze-drying	Microcapsules	Improved survival rate during simulated gastrointestinal tract passage	[102]
Calendula officinalis extract/cosmetic applications	Ionotropic gelation with CaCl_2_ (extrusion or emulsion	Microspheres	The size and entrapment efficiency of microspheres depended on the fabrication method	[103]

**Table 9:** Innovative trends of utilizing gellan gum for the fabrication of responsive systems in biomedical applications.

Innovative Trend	Biomedical Application	References
Smart drug delivery systems	GG has been utilized in the development of advanced drug delivery systems that react to different stimuli, like temperature, pH, or specific ions. These systems can offer the controlled and targeted delivery of therapeutic substances, enhancing the effectiveness of drugs while reducing potential negative reactions	[107,110,111,112]
Temperature-responsive hydrogels	Hydrogels composed of GG have been formulated to demonstrate temperature-sensitive properties, enabling them to transition between sol and gel states based on temperature variations. This characteristic is especially valuable in scenarios where in situ gelation is required, like injectable hydrogels for minimally invasive drug delivery or tissue engineering.	[107,118,119]
Ion-responsive systems	GG can produce gels when exposed to certain ions, like calcium or potassium. This characteristic has been utilized in the creation of ion-responsive systems, which are designed to release drugs or bioactive agents in response to certain ions found in the body.	[72,120]
3D bioprinting and tissue engineering	When combined with other biomaterials, GG has been studied for its potential in 3D bioprinting for tissue engineering. Due to GG’s capability of producing thermoreversible gels, it enables the development of intricate structures with improved mechanical characteristics, ideal for scaffold production.	[113,114,121,122]
Wound healing and dressings	Research has been conducted on hydrogels composed of GG for potential uses in promoting wound healing. These hydrogels offer a moist environment, strong adherence to the wound site, and the potential release of bioactive compounds to improve the healing process. Features can be integrated to cater to particular wound conditions.	[115,116]
Injectable systems for minimally invasive procedures	The thermoreversible gelation property of GG has been utilized to develop injectable systems for minimally invasive procedures. These systems have the capability to be administered in a liquid state and then undergo gelation in situ, which renders them ideal for various applications like tissue augmentation or local drug delivery.	[123,124,125]
Diagnostic applications	GG has been investigated for its use in producing diagnostic devices and biosensors. Utilizing the unique properties of GG, it is possible to develop sensing platforms capable of detecting particular biomolecules or variations in physiological conditions.	[111,112,114,117,126]
Combination with nanoparticles	GG can be combined with nanoparticles, like drug-loaded nanoparticles or imaging agents, to develop multifunctional responsive systems with improved therapeutic or diagnostic capabilities.	[121,127,128]

**Table 10:** Promising future research prospects for researchers and scientists in gellan gum microbial polysaccharides.

Industrial Field	Major Area	New Possibilities for Study and Investigation
Food industry	Functional foods	Investigate the possibility of using it in the production of functional meals that have qualities that are beneficial to patients’ health. GG microbial polysaccharides should be investigated for their potential to be included in functional meals to achieve a number of health advantages, including better gut health and greater nutrient delivery.
	Clean-label solutions and applications	GG is a natural and flexible component that should be investigated for its potential use in clean-label products. The use of GG microbial polysaccharides as natural thickeners, stabilizers, and emulsifiers should be incorporated into the development of clean-label food compositions.
Biopharmaceuticals	Biotherapeutics	Research should be conducted to investigate the use of GG microbial polysaccharides in the creation of biotherapeutics such as vaccinations, monoclonal antibodies, and gene treatments.
	Pharmaceutical excipients	Research should be conducted to determine whether GG microbiological polysaccharides may be used as medicinal excipients with certain functions.
	Tablet formulation	It is important to investigate whether or not GG is suitable for tablet formulations, taking into account its gelling and binding capabilities.
	Oral drug delivery:	GG has the potential to be used in oral medication delivery systems for controlled-release formulations; thus, it should be investigated.
Biomedical applications	Biosensors	When it comes to medical diagnostics, it is important to investigate the possibility of using GG microbial polysaccharides in biosensors. This would enable the identification of certain biomarkers or infections.
	Personalized drug formulations	Through the use of GG-based carriers that are customized to meet the requirements of specific patients, the possibility of individualized medication formulations should be investigated.
	Drug delivery systems	GG, which is biocompatible and has the capacity to harden into gels, has the potential to be investigated as a potential material for drug delivery systems. Advanced drug delivery systems that use micro or nanoparticles derived from GG have the potential to be investigated to achieve the regulated and targeted release of medicinal substances.
	Biocompatible scaffolds	For tissue engineering applications, GG-based scaffolds have the potential to be explored, with a particular emphasis on their structural integrity and their capacity to support cell development.
	Wound healing	GG has the potential to be researched for its use in wound healing applications, maybe as a component in dressings or scaffolds.
Biotechnology	Microbial production optimization	To increase output while simultaneously lowering manufacturing costs, the microbial production process of GG should be optimized.
	Strain improvement	To enhance the effectiveness of the production of GG from *Sphingomonas elodea*, it is recommended that various genetic engineering techniques be investigated.
	Synthetic polysaccharides	To the development of unique GG polysaccharides with specific architectures and functionalities, synthetic biology approaches should be utilized to create microorganisms.
	Genome editing	To achieve precise control over the GG microbial polysaccharide production pathways, it is necessary to utilize sophisticated genome editing technologies.
Environmental applications	Wastewater treatment	It is important to investigate the possibility of using GG in wastewater treatment procedures because of the gel-forming qualities it possesses. Research should be conducted to investigate the possibility of using GG microbial polysaccharides in wastewater treatment processes, such as the biosorption of pollutants or the use of flocculants.
	Bioremediation	In the context of bioremediation applications, it is recommended to investigate the effect that GG plays in facilitating the activities of microorganisms.
	Biodegradable polymers	It is important to conduct more research into the utilization of GG microbial polysaccharides as alternatives to synthetic polymers, with the primary goal of improving their biodegradability and lowering their impact on the environment.
Materials science	Hydrogel applications	An investigation of GG-based hydrogels for a variety of purposes, including medication delivery and tissue engineering, should be conducted.
	Biodegradable films	It is important to investigate the possibility of using GG in the production of biodegradable films for use in packaging.
	Nanostructured materials	An investigation into the fabrication of nanostructured materials via the utilization of GG microbial polysaccharides for uses in nanocomposites, sensors, and electrical devices should be conducted.
	Responsive materials	It is possible to investigate intelligent and responsive materials by adding stimuli-sensitive components into constructions based on GG. This would enable regulated reactions to alterations in the surrounding environment.
Advanced analytical applications	Characterization techniques	The development of sophisticated analytical methods for the accurate characterization of GG and its derivatives should be progressed.
	Molecular studies	To improve both the understanding and the possibilities for application, it is important to investigate the molecular interactions of GG.

## Data Availability

All data reported throughout the manuscript.
